# Editorial: Naturalistic neuroscience — Towards a full cycle from lab to field

**DOI:** 10.3389/fncir.2023.1251771

**Published:** 2023-08-08

**Authors:** Susanne Hoffmann, M. Jerome Beetz, Anna Stöckl, Karen A. Mesce

**Affiliations:** ^1^Department of Behavioural Neurobiology, Max Planck Institute for Ornithology, Seewiesen, Germany; ^2^Department of Behavioural Neurobiology, Max Planck Institute for Biological Intelligence, Seewiesen, Germany; ^3^Department Zoology II, Julius Maximilian University of Würzburg, Würzburg, Germany; ^4^Department of Neurobiology, University of Konstanz, Konstanz, Germany; ^5^Department of Entomology, University of Minnesota, St. Paul, MN, United States

**Keywords:** animal behavior, sensory system, motor control, brain, natural stimuli

Due to the flexible and adaptive nature of the nervous system and to the complexity of information the brain is constantly processing, it is extremely difficult to understand fully the neural mechanisms of behavior. Natural behavioral patterns are often the product of multidimensional integration of sensory information and internal state. To isolate specific features of this complexity under controlled experimental conditions, previous research has often reduced the dimensionality of parameters influencing a behavior. Such a simplified research design is undoubtedly very valuable for testing causal relationships between brain function and behavior. Now that a firm understanding of fundamental neural principles has been established, neuroscience is incorporating these principles into a more natural context in which they have evolved. In order to achieve an ecologically generalizable understanding of how the brain controls behavior, naturalistic contexts at biological, mental and social levels should be considered when designing neuroethological experiments. Recent technological developments, for example, encompassing virtual reality techniques (Madhav et al., 2022), now enable researchers to study the neural mechanisms of behavior under more naturalistic contexts. Within a naturalistic neuroscience framework (Figure 1), it was recently possible to uncover statistics in natural sensory information that match behavioral switches (Bigge et al., 2021), to facilitate the elucidation of how active flight modulates compass representation in the insect brain (Beetz et al., 2022), and even to record brain activity in vocally interacting wild birds that ranged completely free in their natural habitat (Hoffmann et al., 2019).

**Figure 1:**
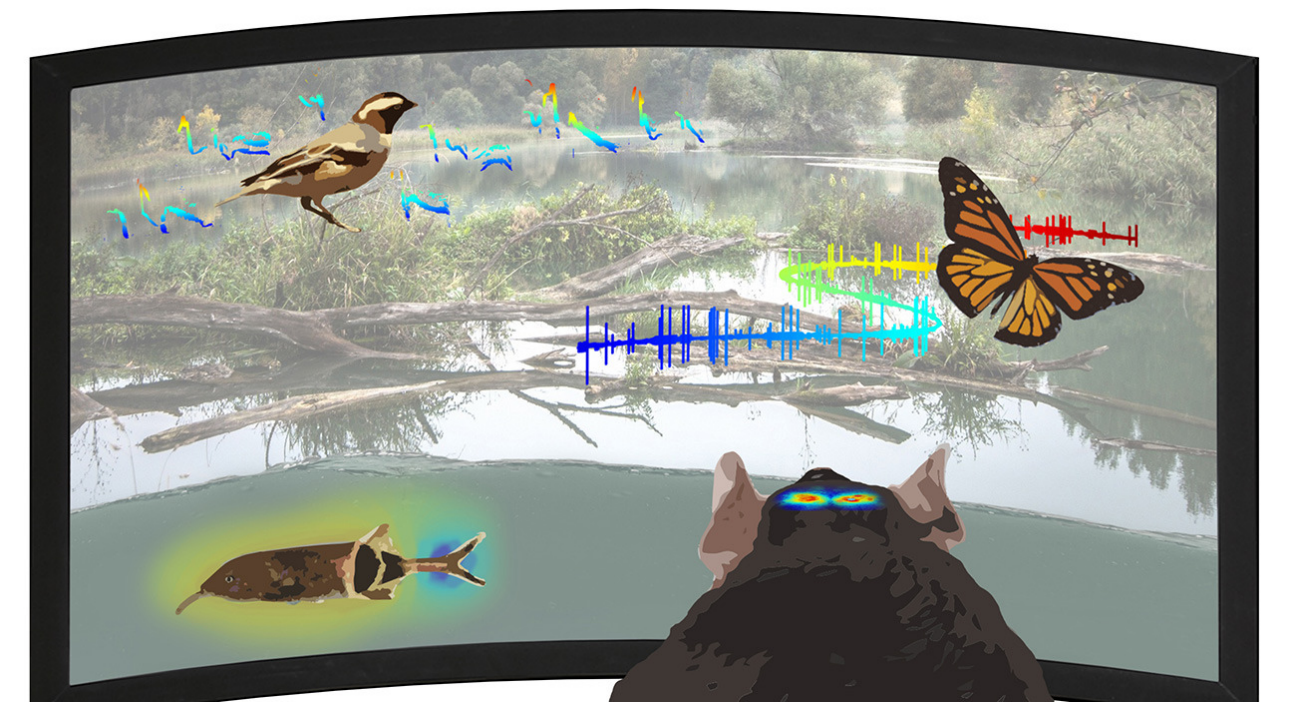
Naturalistic neuroscience - Toward a full cycle from lab to field.

In this Research Topic, 12 articles highlight experimental approaches and technological advancements that consider natural contexts when investigating the neural bases of animal (including human) behavior, and take Naturalistic Neuroscience from the lab to the field. The Research Topic includes eight original research articles, one brief research report, three reviews, and one methods article.

One crucial aspect of naturalistic neuroscience is to successfully characterize the behavior of animals in the wild. Because wave-type electric fish continuously generate electric signals for navigation and social communication purposes, spatio-temporal properties of their electric signals can be used by researchers to localize and follow individual electric fish. In their methods article, Raab et al. introduce an improved algorithm for tracking wave-type electric fish. This algorithm, which is more robust against detection losses than previous approaches, relies on the combined detection of individual-specific spatial and temporal characteristics of electric signals emitted by the fish. The authors successfully tracked individuals of *Apteronotus leptorhynchus* in their natural habitat demonstrating the relevance of their algorithm.

Using a similar approach, Givon et al. studied the spatial behavior of rivulated rabbitfish in their natural reef habitat to pave the way for future research on the neurobiology of free-range navigation. They triangulated the position of individual fish from acoustic recordings of sound signals emitted by tags implanted in the fish. Each individually tracked fish showed a fixed home range within which they perform repeated navigation behavior, which suggests that the neural basis of their navigation behavior could be investigated in natural settings in the near future.

On land, the neurobiology of navigation has been extensively studied in insects. Yilmaz et al. investigated the homing strategies of wild balbyter ants in South Africa to define their relative usage of landmarks and path integration during short distance navigation. The authors conclude that even when foraging in the close vicinity of their nest, balbyter ants benefit from path-integrated vectors to initially define their homing direction.

To study the neural mechanisms of insect navigation, Nguyen et al. recorded intracellularly from the brain of monarch butterflies while presenting different combinations of visual orientation cues. The authors concluded that central complex neurons integrate celestial and terrestrial information, and weigh them flexibly to allow a highly dynamic cue preference during navigation.

The insect's central complex represents a central hub for goal-directed behavior. By recording neural signals from actively hunting praying mantises Wosnitza et al. showed that central-complex neurons can flexibly switch between encoding the mantis's own movement and the movement of potential pray. This study not only demonstrates that the central complex is involved in hunting behavior, but also emphasizes that the insect's behavioral state strongly modulates the tuning of its neurons.

The neurobiology of acoustic behavior is another major research area in neuroethology. In their brief research report, Ferreiro et al. introduce a freely-moving search paradigm to study human auditory perception. Combining live position tracking with wireless sound stimulation, they reintroduced the natural interdependence of an individual's own locomotion and the sensory environment into a laboratory setting.

Avian systems have long been vital to understanding the neural substrates of acoustic-mediated behaviors. By inactivating the auditory brain area NC in female Bengalese finches, Lawley et al. demonstrate the importance of this nucleus for male song preference. Their study suggests the involvement of the female bird's NC in song evaluation and mate choice.

In their review article, Coleman et al. discuss recent findings from studies on sensory cues and motor patterning that are used by songbirds for vocal coordination during duet singing. The authors emphasize that acoustic cues from the respective duet partner are necessary to link the vocal pattern-generating circuit in the brain between two duetting individuals, and also showcase the challenges of recording neural activity in field-based settings.

Bats have also been instrumental in progressing the field of acoustic-mediated behaviors. With their study on the influence of behavioral state on vocal control, Luo et al. demonstrated that experimental conditions have a strong influence on the sound frequency adjustments that are performed by echolocating bats to match the frequency of emitted sounds to their auditory sensitivity. Pratt's roundleaf bats that were naturally flying, and conspecifics that were artificially moved on a pendulum, precisely compensated for motion-induced doppler shifts in returning echoes of their sonar emissions; stationary bats, however, that only listened to echo playbacks did not perform Doppler shift compensation.

How the auditory system of echolocating bats processes naturalistic echo information is reviewed by Beetz and Hechavarría. This review article mainly focuses on different behavioral contexts and their potential to influence the neural processing of echo information. Possible future directions for this area of research, within the framework of naturalistic neuroscience, are discussed at the end of the article.

A huge step in investigating neuroethological mechanisms was the development of biologgers, which are small devices that are carried by animals and record behavioral or physiological data. Gaidica and Dantzer introduce a new implantable biologger, which they designed to study the neurobiology of sleep. The device was tested in laboratory rats and captive squirrels, and showed great potential for long-term EEG recordings in freely behaving animals in laboratory and field-based settings.

Another indispensable technique for naturalistic neuroscientific research is virtual reality (VR), which allows subjects of study to interact with a completely controllable stimulus environment. In his review article, Thurley explains what we understand by VR and why VR is useful for neuroethological research, and describes the technical components needed for naturalistic VR. The review concludes with a discussion of the potential and limitations of VR for naturalistic neuroscience.

In conclusion, we provide a Research Topic of compelling research studies and reviews in our recent Research Topic Naturalistic Neuroscience, which highlight exciting advances in taking neuroscience to the natural context in which circuits and behaviors ultimately operate.

## Author contributions

For the Research Topic, all authors generated a list of potential authors, invited their contributions and handled their manuscripts throughout the peer-review process. The initial draft of this editorial was written by SH and edited by all authors. All authors approved the submitted version of the editorial.
